# A Conundrum of Severe Hypokalaemic Quadriparesis, Acute Kidney Injury, and Lung Involvement as the Initial Presentation of Catastrophic Primary Sjögren’s Syndrome: Is it a New Entity? A Case Report

**DOI:** 10.31138/mjr.040923.acs

**Published:** 2023-09-04

**Authors:** Vishal Mangal, Gaurav Vohra, Sudipt Adhikari, Anil Vasudeva

**Affiliations:** 1 Department of Internal Medicine, Military Hospital Ambala, Haryana, India,; 2 Department of Nephrology, Command Hospital Western Command, Chandimandir, Haryana, India,; 3 Department of Pulmonology, Command Hospital Western Command, Chandimandir, Haryana, India,; 4 Military Hospital Ambala, Haryana, India

**Keywords:** primary Sjögren’s syndrome, catastrophic, distal renal tubular acidosis, hypokalaemia, organising pneumonia, case report

## Abstract

Sjögren’s syndrome (SS) is a systemic chronic autoimmune disorder that classically affects the exocrine glands. Only 15% of the patients with primary SS (pSS) develop extraglandular symptoms involving the lungs, kidneys, joints, nervous system, and skin. Hypokalaemic paralysis is a rare presentation. The most common cause of hypokalaemia is distal renal tubular acidosis. The prevalence of clinically significant lung involvement in pSS is 9-20 %. Primary SS is an indolent disease leading to increased morbidity and poor quality of life. We present a case of a 40-year-old female with severe hypokalaemic paralysis, tubulointerstitial nephritis, and lung involvement as the initial presentation of catastrophic pSS without sicca symptoms. The course of hospitalisation was complicated by ventilator-associated pneumonia. She was managed with broad spectrum antibiotics, five sessions of plasma exchange and alternate-day haemodialysis followed by oral glucocorticoids and intravenous cyclophosphamide. To the best of our knowledge, this is the first case of catastrophic presentation of pSS with a favourable outcome.

## INTRODUCTION

Sjögren’s syndrome (SS) was first described in 1933 by Dr. Henrik Samuel Conrad Sjögren, a Swedish ophthalmologist, as a triad of xerostomia, polyarthritis, and keratoconjunctivitis sicca.^1^ SS is a systemic chronic autoimmune disorder that classically affects the exocrine glands, and only 15% of the patients with primary SS (pSS) develop extraglandular symptoms involving the lungs, kidneys, joints, nervous system, and skin.^2^ SS syndrome predominantly affects middle-aged females in their fifth decade, with a male-female ratio of 1:9.^3^ In the western world, the prevalence of SS is estimated to be 0.04%.^4^ The prevalence of kidney disease in pSS ranges from 4%-7% in European studies compared to much higher rates in Asian countries, with 33.5 % in the Chinese population^5^ and around 50 % in the Indian population.^6^ The renal involvement can present as tubular dysfunction in the form of renal tubular acidosis, Fanconi syndrome, Bartter syndrome, Gitelman syndrome, and nephrogenic diabetes insipidus, nephrolithiasis, tubulointerstitial nephritis, glomerular involvement can present as a nephritic syndrome or nephrotic syndrome with membranoproliferative glomerulonephritis as the most common glomerular lesion.^7^ Hypokalaemic paralysis is a rare presentation; until 2020, only 59 cases have been reported in the literature.^8^ The most common cause of hypokalaemia is distal renal tubular acidosis (dRTA). The prevalence of complete dRTA is estimated to be 5 % compared to 25% for incomplete dRTA.^9^ The prevalence of clinically significant lung involvement in pSS is 9-20 %, whereas subclinical involvement of lungs, as evident on the computed tomographic scan, can be seen in 34-50% of the pSS patients.^10^ We present two cases that presented with severe hypokalaemic paralysis, tubulointerstitial nephritis, and lung involvement as the initial presentation of catastrophic pSS without sicca symptoms. To the best of our knowledge, such a catastrophic presentation of pSS has never been reported in the literature.

## CASE REPORT

**Figure 1.**
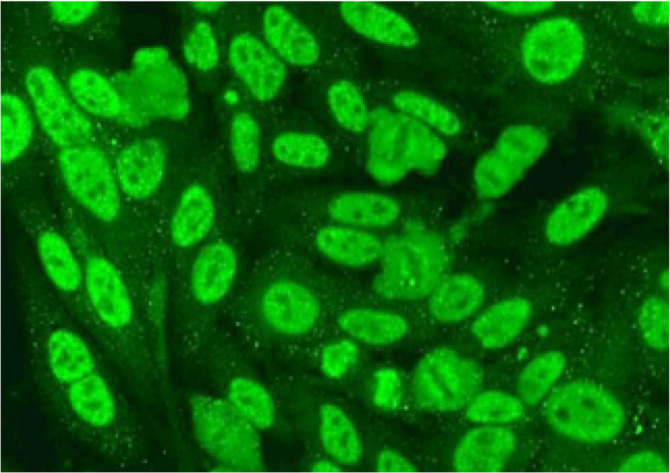
3+ speckled nuclear pattern of antinuclear antibodies by indirect immunofluorescence in the patient.

A 40-year-old female, a known case of primary hypothyroidism on Tablet Levothyroxine, 100 micrograms for the last five years now brought to the emergency department in comatose condition with a history of increased frequency of micturition for the previous five days and gradually worsening weakness of all four limbs of two days duration and two episodes of vomiting since morning. On admission, the patient had a Glasgow coma score of 3/15, with a pulse rate of 70 beats per min, blood pressure of 114/70 mm of Hg, and oxygen saturation of 99 % on oxygen by non-rebreathing mask @ 8 L/min. On examination, she had pallor, and the rest of the systemic examination was essentially normal. Initial laboratory evaluation revealed azotaemia, neutrophilic leucocytosis, anaemia, and severe hypokalaemia (**Table 1**) and combined high anion gap metabolic acidosis with respiratory acidosis along with normal anion gap metabolic acidosis (**Table 2**).The patient was immediately intubated with 7.5mm Internal Diameter oral Polyvinyl chloride cuffed endotracheal tube with pre-medication of 100mcg fentanyl. She was maintained on continuous mandatory ventilation mode with 450ml tidal volume, peak end-expiratory pressure of 5cm, and respiratory rate of 16/min. A right jugular central venous catheter was placed, and injection potassium chloride was administered at the rate of 10 meq per hour. The patient had normal C-reactive protein and negative serum procalcitonin levels. She was managed with fluid resuscitation. The next day patient started moving all four limbs, and potassium supplementation was continued, and because of normal anion gap metabolic acidosis, the possibility of distal renal tubular acidosis was considered. On day three of admission patient developed a fever with increased secretions from the endotracheal tube along with new opacities on the chest radiograph with worsening azotaemia. Subsequently, she developed hypotension. She was started on broad-spectrum antibiotics and inotropes and underwent urgent haemodialysis. She underwent fibreoptic bronchoscopy and broncho-alveolar lavage (BAL) on day five. She underwent high resolution computed tomography of the chest, which revealed multiple areas of consolidation with surrounding ground glass opacities in bilateral lungs suggestive of organising pneumonia with interstitial lung disease. Her autoimmune workup showed 3+ anti-nuclear antibodies (**Figure 1**) with positive SS-A and SS-B (**Table 3**). Her BAL Biofire (BioFire Diagnostics, Utah, USA) showed Acinetobacter Calcoaceticus-Baumannii complex, Klebsiella pneumonia, Proteus spp. Staphylococcus aureus, and Pseudomonas aeruginosa.

**Table 1.** Laboratory parameters.

**Lab Parameters**	**On admission**	**At Discharge**	**Reference range**
Haemoglobin	9.1	9.8	12 to 16 g/dL
Total leucocyte count	35000	11200	4000 to 11000 cells/microL
Platelets	260,000	210000	150000 to 450000 cells/microL
Blood Urea	57	30	8 to 20 mg/dL
Serum Creatinine	2.2	0.9	0.50 to 1.10 mg/dL
Serum Sodium	146	135	136 to 145 mEq/L
Serum Potassium	1.5	3.6	3.5 to 5 mEq/L
Total Bilirubin	0.5		0.3 to 1 mg/dL
AST	85	50	10 to 40 U/L
ALT	60	50	10 to 40 U/L
Serum Calcium	8.7	-	8.6 to 10.2 mg/dL
Total Protein	6.8		5.5 to 9 g/dL
Albumin	3.0	2.3	3.5 to 5.5 g/dL
Globulin	3.8		2.5 to 4.0 g/dL
Urine Glucose	Negative	Negative	Negative
Urine PCR	3490	-	Less than 200mg/g
Urine Routine and microscopic examination	Albumin + RBC:2–3 per HPF Pus cells:6–8 per HPF		

**Table 2.** Arterial blood gas analysis on admission.

**Parameter**	**Patient Value**	**Reference range**
pH	6.770	7.35 to 7.45
PCO_2_	91.2	35 to 45 mm of Hg
Bicarbonate	13.2	21 to 27 mEq/L
Chloride	112	98 to 106 mEq/L
Lactate	0.8	0.7 to 1.8 mEq/L

**Table 3.** Autoimmune workup.

**Laboratory Parameter**	**Value**
C-Reactive Protein	3.6
Procalcitonin	<0.05
T3	0.90
T4	53.07
TSH	25.09
Anti-nuclear antibody by IIF	3+ Nuclear Speckled pattern at dilution of 1:1000
Rheumatoid Factor	Negative
Ds-DNA	Negative
PM-Scl	Negative
U1-nRNP/Sm	Negative
SS-A	Strong Positive
Ro-52	Strong Positive
SS-B/La	Strong Positive
Jo-1	Negative

She underwent an elective tracheostomy on day six because of the expected prolonged intubation. Her antibiotics were changed as per the sensitivity pattern. She was administered five sessions of haemodialysis, and because of active infection, immunosuppression was not contemplated, and she underwent five cycles of plasma exchange on alternate days. Subsequently, the tracheostomy was closed on day 17 of admission.

We established the diagnosis of pSS in our patient in the absence of sicca symptoms as she had severe involvement in two domains of EULAR SS disease activity index questionnaire. Renal involvement in the form of severe hypokalaemia due to dRTA and tubulointerstitial nephritis leading to acute kidney injury requiring haemo-dialysis and lung involvement in the form of organising pneumonia. She scored 4 points out of the five criteria items listed in ACR/EULAR classification criteria for pSS (3 points for Anti-Ro/SSA positivity and 1 point for positive Schirmer test). She was administered oral Prednisolone 0.5 mg/Kg, hydroxychloroquine, and oral potassium supplementation. She was administered injection Cyclophosphamide 500 mg in 500 ml normal saline over 4 hours. The patient was discharged after 29 days of admission. She is on regular follow up.

## DISCUSSION

Currently, the 2016 American College of Rheumatology (ACR) and European League Against Rheumatism (EULAR) classification criteria is used to classify the patients of SS.^11^ The classification criteria are designed to improve the recruitment of patients in clinical trials; however, they cannot be exclusively relied upon in clinical practice to establish the diagnosis of pSS. As per 2016 ACR/EULAR classification criteria, a subject can be classified as pSS if they have at least one sicca symptom pertaining to the eye or oral cavity or positivity of at least one of the domains of ESSDAI questionnaire^12^ plus a score of ≥ 4 obtained from the sum of five objective items. Our patient had a moderate activity in pulmonary domain with clinical and imaging evidence of interstitial lung disease (ILD). She had moderate activity in the renal domain. She had anti-nuclear antibodies positive by indirect immunofluorescence (IIF) with 3+ speckled nuclear patterns and markedly raised anti-SS-A/Ro and anti-SS-B/La antibodies. She also had positive Schirmer’s test with a 4mm length of the moistened strip after 5 minutes.

Hypokalaemia can be seen in 30–47% of patients with pSS.^13^ Mostly it is asymptomatic but rarely can cause paralysis. Our patients had severe hypokalaemia and quadriparesis she presented with respiratory muscle paralysis with respiratory acidosis. The most common cause of hypokalaemia is dRTA. Complete dRTA is characterised by normal anion gap metabolic acidosis and urine pH< 5.5. In incomplete dRTA, serum bicarbonate levels are normal. Our patients had normal anion gap metabolic acidosis and high anion gap metabolic acidosis. The normal anion gap metabolic acidosis was due to complete dRTA leading to severe hypokalaemia, and high anion gap metabolic acidosis was due to acute kidney injury and uraemia. Complete dRTA occurs in only 5% of the patients with pSS.^9^ Our patients had tubulointerstitial nephritis leading to acute kidney injury. She had azotaemia and tubular proteinuria. She required urgent haemodialysis.

The lung manifestations of SS can are divided into three categories: 1) Airway abnormalities leading to cough, bronchial hyper-responsiveness, bronchiolitis, and bronchiectasis. 2) Interstitial lung disease, including non-specific interstitial pneumonia (NSIP), usual interstitial pneumonia (UIP), lymphocytic interstitial pneumonitis (LIP), and organising pneumonitis (OP). 3) Other pulmonary manifestations include pulmonary amyloidosis, lymphoma, pulmonary embolism, and pulmonary hyper-tension.^10^ NSIP is the most common pattern of ILD, with OP constituting only 7% of ILD in SS.^14^ A high titre of anti-SSA is a predisposing factor for developing ILD.^15^ Our patient initially had OP, as she developed hypoxia, new opacities on chest radiograph after 48 hours of intubation. Ventilator associated pneumonia (VAP), by definition, is considered after patient is on ventilator for more than 72 hours. Our patient subsequently developed VAP also.

EULAR recommends using glucocorticoids and cyclophosphamide to manage organ/life-threatening systemic complications involving the lungs, central nervous system, or kidneys.^16^ Our patient had life threatening renal and lung involvement, and the course of hospitalisation was complicated with the development of VAP, and we managed her with six cycles of plasma exchange and alternate day haemodialysis, followed by oral glucocorticoids along with cyclophosphamide 500 mg intravenous. She had a favourable outcome and is currently on regular follow-up.

Hypokalaemic paralysis as the initial presentation of pSS is increasingly being reported.^17^ However, to the best of our knowledge, such a catastrophic presentation of pSS with severe lung and renal involvement with hypokalaemic paralysis causing respiratory muscle weakness in the absence of sicca symptoms has never been reported in the literature.

## ABBREVIATIONS

ACR: American College of Rheumatology
BAL: Bronchoalveolar lavage
dRTA: Distal renal tubular acidosis
EULAR: European League Against Rheumatism
ESSDAI: EULAR Sjögren〉s syndrome (SS) disease activity index
ILD: Interstitial lung disease
IIF: Indirect immunofluorescence
LIP: Lymphocytic interstitial pneumonitis
NSIP: Non-specific interstitial pneumonia
OP: Organising pneumonitis
pSS: Primary Sjögren’s syndrome
SS: Sjögren’s syndrome
UIP: Usual interstitial pneumonia
VAP: Ventilator associated pneumonia
